# Acute Myeloid Leukemia as a Trigger for Hemolytic–Uremic Syndrome

**DOI:** 10.3390/jcm13216468

**Published:** 2024-10-28

**Authors:** Jonas El Bachouti, Anna Domínguez-Guasch, Yolanda Arce, Guadalupe Oñate, Helena Marco, Montserrat Diaz, Lluís Guirado, Roser Torra, Xoana Barros

**Affiliations:** 1 Department of Nephrology, Fundació Puigvert, 08025 Barcelona, Spain; jelbachouti@fundacio-puigvert.es (J.E.B.); adominguezg@fundacio-puigvert.es (A.D.-G.); hmarco@fundacio-puigvert.es (H.M.); mmdiaz@fundacio-puigvert.es (M.D.); lguirado@fundacio-puigvert.es (L.G.); rtorra@fundacio-puigvert.es (R.T.); 2 Department of Pathology, Fundació Puigvert, 08025 Barcelona, Spain; yarce@fundacio-puigvert.es; 3 Department of Hematology, Hospital de la Santa Creu i Sant Pau, 08025 Barcelona, Spain; gonate@santpau.cat

**Keywords:** acute myeloid leukemia, eculizumab, hemolytic uremic syndrome

## Abstract

Acute myeloid leukemia (AML) has not been identified as a cause of secondary hemolytic–uremic syndrome (HUS). This case report describes a woman who developed severe HUS at the time of AML diagnosis and responded favorably to initial treatment with eculizumab, which stabilized her condition and allowed for treatment of the AML. After one year, with stable renal function and genetic studies reported as normal, eculizumab was successfully discontinued. The prompt use of eculizumab was critical to the patient’s survival and improvement in renal function, highlighting the efficacy of early eculizumab treatment in secondary HUS.

## 1. Introduction

Hemolytic–uremic syndrome (HUS) is a condition characterized by a triad of microangiopathic hemolytic anemia, thrombocytopenia, and acute kidney injury.

Although the terminology is changing, three main forms of the disease are proposed: typical HUS, atypical HUS (aHUS), and secondary forms of HUS.

Typical HUS is primarily associated with Shiga toxin-producing Escherichia coli infections, usually affects children after episodes of bloody diarrhea, and is generally self-limited. In contrast, aHUS is associated with dysregulation of the alternative complement pathway due to the presence of antibodies or genetic mutations that affect complement regulation. However, in some cases, these cannot be detected, and aHUS may be a diagnosis of exclusion. The diagnosis of secondary HUS is reserved for cases where an identified trigger is present, such as infections, drugs, autoimmune diseases, transplants, or malignancies. Treatment consists of addressing the underlying cause.

Benefit from the use of C5 blockade has been demonstrated in some forms of secondary HUS, suggesting that dysregulation of the alternative complement pathway may be involved in its pathogenesis [1,2,3]. The differential diagnosis between atypical and secondary HUS remains a significant challenge in clinical practice. Consequently, treatment with C5 inhibitors is recommended in severe cases of secondary HUS that are refractory to therapy for the underlying cause. The potential benefit of its early use in this subgroup of patients still remains uncertain [3,4,5,6].

Oncological and hematological diseases have been described as causes of secondary HUS. However, acute myeloid leukemia (AML) has not been previously identified as a potential trigger for this condition.

AML is a type of cancer that affects myeloid progenitor cells, characterized by the uncontrolled proliferation of abnormal blast cells. This expansion interferes with the normal production of blood cells in the bone marrow, leading to its failure and preventing the normal production of red blood cells, white blood cells, and platelets, resulting in pancytopenia. Complications of AML can be attributed to both the disease itself and to the treatments employed. A common complication is bleeding, which can be caused by thrombocytopenia, infiltration of malignant cells in the liver, or disseminated intravascular coagulation (DIC) [7,8,9].

The pancytopenia resulting from leukemia or DIC may present a challenge in differential diagnosis with HUS due to overlapping clinical and laboratory findings, particularly concerning anemia and thrombocytopenia. Careful analysis of the underlying mechanisms and additional diagnostic tests, such as the identification of blasts in leukemia, coagulation abnormalities in DIC, and the presence of microangiopathic hemolytic anemia in HUS, are essential for a correct diagnosis [7,8,9,10].

This report discusses the first published case of secondary HUS attributed to AML. Additionally, it underscores the importance of early eculizumab administration in this secondary form of HUS.

## 2. Clinical Case

We present a 58-year-old female from Poland, ex-smoker (20 packs/year), with a medical history of dyslipidemia and ischemic heart disease, with normal previous renal function. She presented to the hospital emergency department with febrile syndrome, reaching a body temperature of 40 °C, associated with a dry cough without other symptoms. The patient was diagnosed with right lower lobe pneumonia due to Legionella pneumophila and started on antibiotic treatment with levofloxacin. Laboratory tests showed pancytopenia with a hemoglobin (Hb) of 9.4 g/dL, leukocytes of 0.81 × 10^9^/L, and platelets of 66 × 10^9^/L. The peripheral blood smear showed 8% blasts, suggesting a diagnosis of acute leukemia. On admission, the patient had normal renal function, with a serum creatinine of 0.86 mg/dL.

During her hospitalization, a bone marrow aspiration was performed. AML was diagnosed with nucleophosmin-1 (NPM1) mutations and dual activating mutations in FMS-like tyrosine kinase 3 (FLT3-ITD and FLT3-TKD).

Following stabilization of her pneumonia, the patient developed oliguria, with grade 3 acute kidney injury being detected with a serum creatinine of 3.7 mg/dL and hemodialysis requirement due to a positive fluid balance. In addition, increased blood pressure was noted, and analyses showed signs of peripheral hemolysis with increased LDH of 3493 IU/L (N: 105 to 333 IU/L), the presence of schistocytes, undetectable haptoglobin, and a negative direct Coombs test. The study was extended to include testing for ADAMTs13, which resulted in a normal range of 51%, and a stool culture, with a negative result for Shiga toxin-producing Escherichia coli.

A series of complementary tests were conducted (immunity study, cobalamin metabolism, blood cultures, serologies for human immunodeficiency virus (HIV), hepatitis C virus (HCV) and B virus (HBV), and cytomegalovirus (CMV), and even microbiological study in bronchoalveolar lavage and transthoracic echocardiogram), ruling out other potential causes of secondary HUS and suggesting that the condition was probably linked to her pneumonia or leukemia. Notably, the patient was in remission from pneumonia and did not require oxygen therapy when hemolysis started and subsequently worsened, indicating that uncontrolled leukemia may have been the triggering factor.

**Figure 1:**
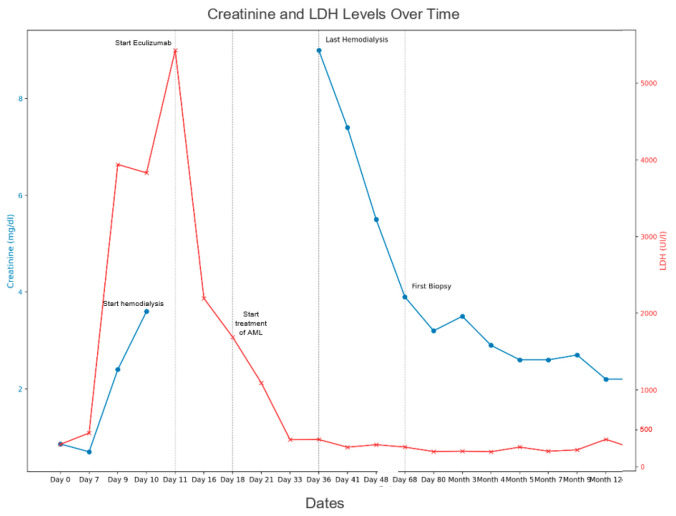
Evolution of kidney function measured by plasma creatinine. The most important events and treatment received are reflected.

At the time of diagnosis, plasma exchange was considered for treatment, as it has traditionally been the standard initial approach for secondary HUS. However, the severity of the patient’s condition, coupled with a paradigm shift in the management of HUS, led to a consensus with the hematology team that early initiation of eculizumab was the most appropriate course of action. She was started on eculizumab 900 mg per week for 4 weeks, along with antibiotic prophylaxis with amoxicillin and the required vaccination. Following the introduction of eculizumab, the hemolysis stabilized within one week, enabling the initiation of leukemia treatment the subsequent week (Figure 1). Given the patient’s situation, she was not a candidate for intensive chemotherapy, and it was decided to treat the AML with the regimen for unfit patients (azacitidine and venetoclax). The patient showed progressive improvement in hemolysis and diuresis recovery with permanent discontinuation of hemodialysis one month after the onset of symptoms. The first bone marrow analysis showed complete remission of AML three months after chemotherapy initiation.

**Figure 2:**
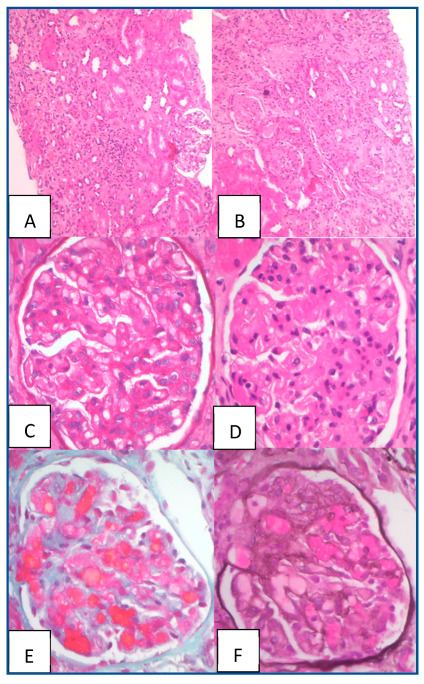
Pathological anatomy of the first renal biopsy with signs of TMA. Images (**A**,**B**) with hematoxylin and eosin and PAS staining: Interstitial fibrosis of 40% of the cortical tissue associated with tubular atrophy and a mild chronic inflammatory infiltrate. Images (**C**,**D**): Ectasia and capillary congestion, associated with focal mesangiolysis. Images (**E**,**F**) with Masson trichrome and silver staining: Highlight the ectasia and capillary congestion.

During outpatient follow-up after hospital discharge, the patient continued treatment with eculizumab 1200 mg every two weeks and chemotherapy. Two months later, there were no signs of hemolysis, and the estimated glomerular filtration rate (eGFR) using the CKD-EPI-creatinine equation was stable at 20 mL/min/1.73 m^2^. In order to guide eculizumab treatment, we decided to perform a renal biopsy. The pathology study reported 18 glomeruli with signs of acute thrombotic microangiopathy (TMA) with moderate chronic changes (Figure 2).

Given the presence of acute TMA lesions in the biopsy, we opted to continue eculizumab treatment. In the following months, genetic studies did not identify any pathogenic variants associated with HUS (CFH, CD46, CFI, CFB, C3, THBD, DGKE, CFP, and ADAMTS13). However, the patient was found to be heterozygous for both CFH and MCP risk haplotypes. Sequential bone marrow analyses confirmed remission of the AML. However, eGFR remained stable at 20 mL/min/1.73 m^2^, exhibiting no improvement, and C5b9 on endothelial cell culture remained elevated in the patient’s laboratory tests, prompting a cautious approach in determining the optimal timing for discontinuing eculizumab.

Finally, after 11 months of treatment, a new renal biopsy was performed, reporting 17 glomeruli with chronic changes, but no evidence of acute TMA. With this information, eculizumab was successfully tapered to complete withdrawal. Twelve months after discontinuation of eculizumab, the patient exhibited stable renal function (eGFR = 25 mL/min/1.73 m^2^) without hypertension, urinary abnormalities, or evidence of peripheral hemolysis. Regarding AML, the patient remains in complete response, with undetectable residual disease two years after treatment.

## 3. Discussion

This case illustrates that AML may act as a trigger for HUS and highlights the importance of early eculizumab treatment.

Although hematological neoplasms such as lymphomas and myelomas have been associated with TMA, AML has not been documented as a cause of secondary HUS.

Our literature search identified a case of TMA secondary to AML, which was classified as thrombotic thrombocytopenic purpura (TTP) rather than HUS [11]. This distinction is significant, as TTP and HUS share common features like microangiopathic hemolytic anemia and thrombocytopenia, yet they differ in their underlying pathogenic mechanisms and treatment strategies. TTP is primarily driven by a deficiency in the ADAMTS13 enzyme, resulting in the formation of large von Willebrand factor multimers. In contrast, HUS, particularly atypical HUS (aHUS), is often associated with complement dysregulation.

Another noteworthy case from 2013 described a patient with acute promyelocytic leukemia (APL), a subtype of AML, who developed TMA [12]. This patient underwent plasmapheresis, a common treatment for TMA, without the concomitant use of a complement inhibitor. Unfortunately, the patient’s condition progressed to chronic kidney failure, requiring long-term hemodialysis.

In the present case, AML was identified as the trigger for HUS, likely in the context of an underlying genetic predisposition, since the patient was a carrier of CFH and MCP risk haplotypes. She presented clinical improvement and sustained survival after early initiation of eculizumab, followed by leukemia treatment and disease remission.

The cases mentioned above highlight the need to distinguish between different types of TMA in the context of hematologic malignancies and to implement early, targeted interventions. These conditions present significant challenges in the diagnostic process. For instance, various confounding factors can delay the recognition of hemolysis in hematologic patients, as anemia and thrombocytopenia may also arise from hematologic pancytopenia or be induced by administered treatments. Furthermore, once hemolysis is detected, commonly used tools such as the PLASMIC score may provide limited utility, leading to potential misinterpretation and diagnostic confusion.

We need more comprehensive research into the role of complement dysregulation in cancer-related TMAs and the potential benefits of C5 blockade in such settings. This benefit should also be investigated in other forms of secondary HUS to assess whether C5 blockade should be initiated at the onset of TMA rather than later, after confirmation of refractoriness to the etiological treatment.

In this aspect, the experience exposed in this report is particularly enlightening. While we cannot ascertain how the patient would have responded to traditional treatments such as corticosteroids and plasmapheresis, the severity of her condition and the paradigm shift in the management of HUS led to the choice of early eculizumab, which yielded a favorable response. Notably, despite this early intervention, the patient exhibited chronic TMA lesions in her kidney biopsy, and improvement in eGFR did not exceed 25 mL/min/1.73 m^2^. This observation raises the hypothesis that renal endothelial damage may have occurred prior to the onset of hemolysis, which may not have been detected earlier by the decline in renal function.

Determining the appropriate time to discontinue C5 blockers remains a challenge. The high cost of the drug, the implications of long-term use, and potential side effects require careful consideration. Lifelong C5 blockade is no longer a universally applicable paradigm for all patients with aHUS, particularly in the secondary forms [7].

In the case described, our treatment approach was guided by histopathological findings. As long as signs of active TMA were present in the biopsy, treatment with eculizumab was maintained. It was surprising to observe signs of acute endothelial damage in the first renal biopsy, conducted two months after treatment initiation. Although there are no previous data on eculizumab in this setting, we believed it was essential to maintain treatment in the presence of these acute TMA lesions, until their resolution. The duration of treatment following the resolution of acute lesions remains to be determined.

Biomarkers of complement terminal pathway activation, such as soluble C5b9 or C5b9 on endothelial cell culture, may help guide this decision, but more research is needed, especially regarding their use for follow-up monitoring [7,13,14,15].

In this case, the decision to discontinue eculizumab was supported by the repeated renal biopsy, which revealed no evidence of active TMA.

## 4. Conclusions

This case highlights the importance of recognizing that secondary HUS is often associated with complement dysregulation, which implies a shift in disease management strategies.

Early use of eculizumab can significantly improve outcomes in patients with AML-related HUS, allowing for successful treatment and discontinuation of dialysis.

Further research is needed on the role of early treatment with complement inhibitors in secondary HUS, as well as to better understand when to stop eculizumab and how to effectively monitor these patients.

## Data Availability

Data are contained within the article.
